# Feasibility and Safety of Small Bowel Capsule Endoscopy in Very Early-Onset Inflammatory Bowel Disease: A Multi-Institutional Study

**DOI:** 10.1093/ibd/izaf144

**Published:** 2025-07-08

**Authors:** Shin-ichiro Hagiwara, Hirotaka Shimizu, Ryusuke Nambu, Keisuke Jimbo, Emiri Kaji, Takuya Nishizawa, Fumihiko Kakuta, Itaru Iwama, Takashi Ishige, Takahiro Kudo, Katsuhiro Arai

**Affiliations:** Department of Gastroenterology, Nutrition, and Endocrinology, Osaka Women’s and Children’s Hospital, Osaka, Japan; Division of Gastroenterology, Center for Pediatric Inflammatory Bowel Disease, National Center for Child Health and Development, Tokyo, Japan; Department of Pediatrics and Adolescent Medicine, Juntendo University Graduate School of Medicine, Tokyo, Japan; Department of Gastroenterology and Hepatology, Saitama Children’s Medical Center, Saitama, Japan; Department of Pediatrics, Juntendo University Faculty of Medicine, Tokyo, Japan; Department of Pediatrics, Osaka Medical and Pharmaceutical University, Osaka, Japan; Department of Pediatrics, Gunma University Graduate School of Medicine, Maebashi, Japan; Department of General Pediatrics and Gastroenterology, Miyagi Children’s Hospital, Sendai, Japan; Department of Gastroenterology and Hepatology, Saitama Children’s Medical Center, Saitama, Japan; Department of Pediatrics, Gunma University Graduate School of Medicine, Maebashi, Japan; Department of Pediatrics, Juntendo University Faculty of Medicine, Tokyo, Japan; Division of Gastroenterology, Center for Pediatric Inflammatory Bowel Disease, National Center for Child Health and Development, Tokyo, Japan

**Keywords:** children, patency capsule, VEO-IBD

## Abstract

**Background:**

Small bowel capsule endoscopy is a valuable modality for evaluating small intestinal lesions in children with inflammatory bowel disease. However, its application for very early-onset inflammatory bowel disease remains insufficiently documented. This study investigated the feasibility and safety of small bowel capsule endoscopy for very early-onset inflammatory bowel disease.

**Methods:**

A cohort of patients with very early-onset inflammatory bowel disease who underwent small bowel capsule endoscopy at under 6 years of age between January 2013 and December 2022 at 7 Japanese pediatric centers was included. Their medical records were retrospectively reviewed for actual procedures, safety, and test results.

**Results:**

Eighty-two patients (21 with ulcerative colitis, 25 with Crohn’s, 31 with inflammatory bowel disease—unclassified, 4 with monogenic inflammatory bowel disease, and 1 with intestinal Behçet’s disease) were enrolled, and 104 small bowel capsule endoscopies (median age, 3.8 years; median body weight, 13.0 kg) were analyzed. All capsules were deployed endoscopically, mostly using delivery devices (95%). Gastrointestinal patency was assessed in 95% of procedures, most commonly using patency capsules (70%). Abnormal small bowel findings were observed in 42% of patients, with aphthae being the most common (34%), followed by ulcers (18%). No serious complications, including small intestinal retention and perforation, were reported.

**Conclusions:**

Small bowel capsule endoscopy for very early-onset inflammatory bowel disease is feasible with diagnostic utility, and it can be performed safely with appropriate evaluation using patency capsules.

**Key Messages**

**What is already known?**

In the diagnosis of pediatric inflammatory bowel disease, small bowel evaluation using small bowel capsule endoscopy or magnetic resonance enterography is recommended for cases other than those of typical ulcerative colitis.

**What is new here?**

In very early-onset inflammatory bowel disease (VEO-IBD) patients under 6 years of age, small bowel capsule endoscopy could be safely performed with appropriate gastrointestinal patency evaluation, allowing the assessment of small bowel lesions that might have been previously overlooked.

**How can this study help patient care?**

The use of small bowel capsule endoscopy allows accurate assessment of small bowel lesions in VEO-IBD patients and may contribute to determine the extent of intestinal involvement and treatment strategies.

## Introduction

Endoscopic evaluation of the gastrointestinal tract is essential for the diagnosis and follow-up of pediatric inflammatory bowel disease (pIBD). The Revised Porto Criteria recommends both upper gastrointestinal endoscopy and colonoscopy when pIBD is suspected.^1^ Unless endoscopic findings indicate typical ulcerative colitis (UC), small bowel capsule endoscopy (SBCE) or magnetic resonance enterography (MRE) is recommended for the observation of small bowel lesions. However, performing SBCE or MRE in patients with very early-onset inflammatory bowel disease (VEO-IBD), diagnosed before 6 years of age, is particularly difficult due to their smaller body size.^2^ Consequently, the observation of small bowel lesions in patients with VEO-IBD remains highly challenging. With the advent of SBCE, it is now possible to observe the small intestinal mucosa non-invasively. SBCE has become a crucial modality for the diagnosis and follow-up of IBD, including Crohn’s disease (CD).^1,3,4^ However, there are significant challenges in performing SBCE in younger children because of the general requirement to swallow the capsule and the risk of retention in their relatively narrow intestinal tract.^5^

Although several studies have demonstrated the feasibility of SBCE in children under 6 years of age—many of whom may fall within the VEO-IBD category—these studies did not specifically focus on patients formally diagnosed with VEO-IBD or systematically evaluate the procedure and safety of SBCE in this unique subgroup.^6–8^

The aim of this multi-institutional study was to clarify the feasibility of SBCE for the diagnosis and follow-up of VEO-IBD. The findings of this study may allow a detailed assessment of the extent of gastrointestinal involvement and mucosal activity in the small intestine using SBCE in VEO-IBD, potentially guiding accurate diagnostic and therapeutic strategies.

## Materials and Methods

### Study Design

This multi-institutional retrospective case series included patients with VEO-IBD who underwent SBCE between January 2013 and December 2022 at 7 pediatric centers in Japan. All patients with VEO-IBD who underwent SBCE within the specified timeframe were included. Patients aged 6 years or older who underwent SBCE for VEO-IBD were excluded from the study. Medical records of the included patients were reviewed and analyzed. Certified pediatric gastroenterologists at each institution assessed the small bowel findings.

The collected data included age, sex, height, body weight, diagnoses, methods of SBCE placement, types of anesthesia and devices used for CE placement, approaches for assessing gastrointestinal patency, SBCE-related adverse events, and small bowel findings.

### Definitions

UC, CD, and inflammatory bowel disease—unclassified (IBD-U) were diagnosed based on endoscopic images and pathological findings using the Revised Porto Criteria.^1^ If monogenic IBD was suspected based on medical history, family history, and blood test results, genetic testing was conducted at the discretion of each center.^9^ Cases of IBD-U identified as being caused by a monogenic etiology were classified as monogenic IBD.

### Ethical Considerations

This study was approved by the Ethics Committee of Osaka Women’s and Children’s Hospital (registration number 1607) and by the ethics committees of the other participating centers. Consent was obtained through the study website, which included provisions for opting out of the study.

### Statistical Analyses

Results are presented using descriptive statistics, including arithmetic medians and ranges, to summarize demographic data and procedural details.

## Results

### Patient Characteristics

Between January 2013 and December 2022, 2259 SBCEs were performed across seven institutions. A total of 104 procedures were performed in 82 patients, 44 of whom were male. In this study, all SBCEs were performed using PillCam SB2 or SB3 (Medtronic). The dimensions of the capsule are 26.2 mm (length) × 11.4 mm (diameter). Patient characteristics are shown in Table 1. The median age was 3.8 years, with ages ranging from 1.5 to 5.9 years. The median height was 93.9 cm, with a range of 72.0-117.0 cm. The median body weight was 13.0 kg, with a range of 9.0-21.0 kg. The patients were diagnosed with UC (21 cases), CD (25 cases), IBD-U (31 cases), monogenic IBD (4 cases), or intestinal Behçet disease (1 case). Four monogenic IBD included 2 XIAP deficiency, trisomy 8, and chronic nonspecific multiple ulcers of the small intestine (CEAS). The primary purpose of SBCE was diagnosis, accounting for 71 cases, whereas in an additional 33 cases, SBCEs were conducted for follow-up assessments.

**Table 1.** Patient characteristics

Subjects, *n*	82 (Male 44)
Procedures, *n*	104
Age, year (median, range)	3.8 (1.5-5.9)
Height, cm (median, range)	93.9 (72.0-117.0)
Body weight, kg (median, range)	13.0 (9.0-21.0)
Diagnosis	
Ulcerative colitis, *n*	21
Crohn’s disease, *n*	25
IBD-U, *n*	31
Monogenic IBD	4
Intestinal Bechet’s disease, *n*	1
Ulcerative colitis (*n* = 21)	
Extent E1, E2, E3, E4	0, 0, 2, 19
Severity S0 (PUCAI < 65), S1 (PUCAI ≥ 65)	13, 8
Crohn’s disease (*n* = 25)	
Disease location	
L1 + L4ab	1
L2, L2 + L4a	6, 3
L3, L3 + L4a, L3 + L4b, L3 + L4ab	3, 2, 2, 5
L4b, L4ab	1, 2
Behavior	
B1, B2, B3, B23, P	25, 0, 0, 0, 9
IBD-U (*n* = 31)	
UC type	8
CD type	23
Purpose for SBCE	
Diagnosis, *n*	71
Follow-up, *n*	33

Abbreviations: CD, Crohn’s disease; IBD-U, inflammatory bowel disease—unclassified; PUCAI, Pediatric Ulcerative Colitis Index; SBCE, small bowel capsule endoscopy; UC, ulcerative colitis.

### SBCE Insertion Method

This study included 104 SBCEs, all performed using a gastroscope (Table 2). Regarding anesthesia methods, 67 cases underwent the procedure under general anesthesia and 37 underwent the procedure under intravenous sedation. The SBCE was placed in the duodenum in 99 cases and in the stomach in 5 cases. The devices used for insertion included the AdvanCE capsule endoscope delivery device (US Endoscopy Inc.) in 99 cases, and a retrieval net in 5. In 65 cases, the procedure was performed along with colonoscopy, whereas the remaining 39 cases underwent SBCE alone.

**Table 2.** Insertion method of SBCE

Insertion method of SBCE	
Using gastroscope, *n*	104
Swallow, *n*	0
Anesthesia	
General anesthesia, *n* (%)	67 (64.4)
Intravenous sedation, *n* (%)	37 (35.6)
SBCE placement site	
Stomach, *n* (%)	5 (4.8)
Duodenum, *n* (%)	99 (95.2)
Device for insertion	
AdvanCE, *n* (%)	99 (95.2)
Retrieval net, *n* (%)	5^b^(4.8)
Performed in conjunction with colonoscopy, *n* (%)	65 (62.5)
Only SBCE with EGD, *n* (%)	39 (37.5)
Preparation, *n* = 39^a^	
No preparation, *n* (%)	8 (20.5)
Magnesium citrate (Magcolol P), *n* (%)	28 (71.8)
Polyethylene glycol solution (Niflec), *n* (%)	2 (5.1)
Sodium picosulfate powder (PicoPrep), *n* (%)	1 (2.6)
Picosulfate, *n* (%)	5 (12.8)
Low-residue diet, *n* (%)	3 (7.7)

Abbreviations: EGD, esophagogastroduodenoscopy; SBCE, small bowel capsule endoscopy.

^a^Cases without concurrent colonoscopy.

^b^The combination of a retrieval net and a snare was employed in 1 case.

Of the 39 cases without concurrent colonoscopy, 8 had no preparation and 28 used magnesium citrate, followed by other medications or a low-residue diet.

### Evaluation of Intestinal Patency Reported to be Evaluated

Intestinal patency was evaluated in 99 cases (95.2%) (Table 3). Among the 99 cases, the methods included a patency capsule (PC) using a scope in 69 cases (69.7%) and PC by swallowing in 1 case (1%). In the 29 cases in which PCs were not used, abdominal ultrasonography (AUS) was most commonly used to evaluate intestinal patency (72.4%). Sixteen cases were evaluated for gastrointestinal patency using only AUS, but this method was limited to only 3 facilities.

**Table 3.** Evaluation of intestinal patency

Evaluation of intestinal patency	
Yes, *n* (%)	99 (95.2)
No, *n* (%)	5 (4.8)^a^
Method of patency judgment (*n* = 99)	
Patency capsule (using scope), *n* (%)	69 (69.7)
Patency capsule (swallow), *n* (%)	1 (1.0)
AUS, *n* (%)	22 (22.2)
CT, *n* (%)	6 (6.1)
MRE, *n* (%)	1 (1.0)
Small bowel contrast study, *n* (%)	6 (6.1)
Cases in which patency capsules were not used	29
AUS alone, *n*	16^b^
AUS and CT, *n*	4
AUS and small bowel contrast study, *n*	1
CT alone, *n*	2
MRE alone, *n*	1
Small bowel contrast study alone, *n*	5

Abbreviations: AUS, abdominal ultrasonography; CT, computed tomography; MRE, magnetic resonance enterography.

^a^Five cases had previously undergone patency capsule and capsule endoscopy examinations, which were completed safely.

^b^Only 3 facilities evaluated gastrointestinal patency using AUS alone.

### Summary of Adverse Events and SBCE Findings

Complete observation of the entire small intestine was achieved in 100 (96.1%) patients (Table 4). Of the remaining 4 patients, 3 could not undergo a complete observation of the entire small intestine due to battery depletion, and 1 had the capsule retained in the stomach. Severe adverse events, such as retention in the small intestine, bleeding, or perforation, were not reported. However, in 1 case, the capsule failed to pass naturally through the rectum and had to be retrieved endoscopically. Small bowel lesions were observed in 42.7% of patients, with aphthae being the most prevalent, accounting for 34.1% of the patients. For CD, abnormal small bowel findings were observed in 48% of the patients, with aphthae being the most common finding, followed by ulcers. Similarly, for IBD-U, 48.4% of patients showed abnormal small bowel findings, with aphthae identified as the most common feature in 41.9% of patients.

**Table 4.** SBCE adverse events and small bowel lesions

Total small intestine observation, *n* (%)	100 (96.1)			
Small bowel passage time (min), median (range)	297 (112-764)			
Adverse events				
Small bowel retention, *n*	0			
Rectal retention, *n*	1^*^			
Perforation, *n*	0			
Bleeding, *n*	0			

	All patients, *n* = 82	CD, *n* = 25	IBD-U, *n* = 31	UC, *n* = 21
Small bowel lesions, *n* (%)	35 (42.7)	12 (48)	15 (48.4)	3 (14.3)
Bleeding, *n* (%)	4 (4.9)	2 (8.0)	2 (6.5)	0 (0)
Aphtha, *n* (%)	28 (34.1)	9 (36)	13 (41.9)	3 (14.3)
Ulcers, *n* (%)	15 (18.3)	6 (24)	2 (6.5)	2 (9.5)
Longitudinal ulcers, *n* (%)	2 (2.4)	2 (8.0)	0 (0)	0 (0)
Cobblestone appearance, *n* (%)	0 (0)	0 (0)	0 (0)	0 (0)
Stenosis, *n* (%)	0 (0)	0 (0)	0 (0)	0 (0)

Abbreviations: CD, Crohn’s disease; IBD-U, inflammatory bowel disease—unclassified; SBCE, small bowel capsule endoscopy; UC, ulcerative colitis.

^a^Necessitated capsule retrieval through endoscopy.

### Impact of SBCE on Clinical Management

Among the 71 cases in which SBCE was performed for diagnostic purposes, it was reported to have contributed to diagnosis in 65 cases. In the remaining 6 cases where SBCE was deemed not contributory, there were 5 cases of IBD-U and 1 case of monogenic IBD. Among the 33 cases in which SBCE was performed for follow-up purposes, SBCE was considered to have contributed to clinical management in 31 cases. In the 2 cases where SBCE was noncontributory, 1 involved gastric retention of the capsule, preventing small bowel observation, and the other was a case of monogenic IBD (CEAS).

## Discussion

To the best of our knowledge, this is the first report on the safety and feasibility of SBCE for the diagnosis and follow-up of VEO-IBD.

Our study revealed that all patients with VEO-IBD underwent capsule placement using an upper gastrointestinal endoscope. Importantly, there were no cases of capsule retention in the small intestine when gastrointestinal patency was assessed. In small bowel monitoring of VEO-IBD by SBCE, 42% of patients had abnormal small bowel findings, the most common of which were aphthous lesions.

Many studies have reported the usefulness of pediatric SBCE, starting with a case report in 2004, in which capsule endoscopy was performed on a 3-year-old boy with chronic anemia, leading to a diagnosis of small bowel hamartomatous polyps.^6,10,11^ This landmark case highlights the potential of SBCE in diagnosing small bowel pathologies in young children, even in challenging clinical scenarios. Since then, numerous studies have demonstrated its diagnostic accuracy and safety in pediatric populations, particularly in conditions such as obscure gastrointestinal bleeding and suspected CD.

Although there is 1 report of a 4-year-old patient who was able to swallow the capsule,^7^ it is generally considered possible for patients to swallow the capsule from 8 to 9 years of age onward.^10,12^ In younger patients, swallowing the capsule is nearly impossible, necessitating its placement in the stomach or duodenum using both an upper gastrointestinal endoscope and a delivery device, such as the AdvanCE capsule endoscope delivery device.^13^ According to previous reports, the minimum body weight required for undergoing SBCE is 7.9 kg.^14^ In this study, the minimum body weight was 9.0 kg. Based on previous reports and the results of this study, the minimum weight at which capsule endoscopy can be considered is approximately 8-9 kg. This body weight may serve as a practical criterion for determining the feasibility of capsule endoscopy in patients with VEO-IBD.

If the patient is unable to swallow the capsule or if SBCE is performed in conjunction with upper or lower gastrointestinal endoscopy, the capsule should be placed endoscopically in the stomach or duodenum. In particular, to prevent the capsule from failing to pass through the pylorus and hindering small bowel observation, placement of the capsule in the duodenum is preferred. Younger patients with VEO-IBD require general anesthesia or intravenous sedation. In this study, approximately two-thirds of patients had a capsule placed under general anesthesia. Considering body size and airway management, general anesthesia is a likely choice for smaller children. However, if a PC is used prior to SBCE, it may require general anesthesia or intravenous sedation twice within a short period. Therefore, careful consideration of its indication is essential.

The PC is a useful and noninvasive tool for evaluating intestinal patency in elder children.^15,16^ However, there have been no reports on the tolerability or usefulness of PCs in younger children. In this study, a PC was the most frequently used device. Similar to SBCE, PC requires endoscopic placement, necessitating two separate endoscopic procedures for patients with VEO-IBD. In the present study, AUS has been used in some institutions to evaluate intestinal patency; however, its limitations should be carefully considered. For example, we encountered a case of VEO-IBD where a PC was retained in the ileocecal region, causing temporary intestinal obstruction, despite the patient weighing 10 kg and having no evidence of stenosis on AUS. Therefore, it is strongly recommended to assess intestinal patency using PCs in small-bodied VEO-IBD patients to minimize the risk of requiring surgical intervention due to SBCE retention.

Gastrointestinal retention is the most critical complication of SBCE. The risk of capsule retention, which can be caused by small bowel stenosis, is particularly high in CD. A study conducted before the widespread use of PC reported a retention rate of 2.2% in pediatric CD^17^; a 2020 meta-analysis found a retention rate of 1.64% in pediatric CD, which was lower than in adults.^18^ This may be due to the shorter duration of CD in children and the presence of fewer stenotic lesions. In VEO-IBD, the pylorus and ileocecal valve are relatively narrow, and the small intestinal diameter is small, increasing the possibility of difficult passage even in the absence of strictures. This population is at high risk for capsule retention, requiring careful assessment of gastrointestinal patency and careful consideration of the indication for capsule endoscopy.

Several studies have shown that the CD-type VEO-IBD is the most common, with the colon-limited (L2) type being the most dominant form.^19,20^ Regarding the prevalence of small bowel lesions, previous reports on CD-type VEO-IBD have described L3 (ileocolonic) involvement in approximately 20% and L4b (upper disease distal to the ligament of Treitz and proximal to the distal third of the ileum) in 13% of cases.^20,21^ In contrast, the present study demonstrated that 24% of CD cases had isolated L2 (colonic) involvement, while the majority exhibited involvement of the small intestine. Given that previous studies assessed small bowel lesions using modalities other than capsule endoscopy, there is a possibility that the prevalence of small bowel involvement was underestimated. SBCE is recognized for its superior diagnostic accuracy compared with MRE or computed tomography enterography (CTE),^22^ making it a valuable tool for a more precise evaluation of the lesion extent in VEO-IBD.

In the evaluation of small bowel involvement in pediatric IBD, especially in the VEO-IBD population, the choice of diagnostic modality must balance sensitivity, safety, and feasibility. SBCE has been reported to be superior to MRE in detecting superficial mucosal activity of the small intestine and proximal-medium small bowel lesions in adult CD.^23,24^ In pediatric ileal CD, MRE demonstrated a sensitivity and specificity of approximately 70% for the detection of endoscopic ulcers.^25^ In contrast, MRE and CTE provide important information on transmural inflammation, bowel wall thickening, and extra-luminal complications.^24,26^ However, their utility may be limited in VEO-IBD due to technical challenges such as motion artifacts, suboptimal spatial resolution in very small patients, and the frequent need for sedation. Furthermore, the ionizing radiation associated with CTE is a significant concern. This is particularly relevant when repeated imaging is anticipated. In this context, SBCE offers a radiation-free, relatively well-tolerated, and highly sensitive method for mucosal evaluation in VEO-IBD, although it may require intestinal patency testing and specialized equipment for capsule delivery in very young children. Therefore, while MRE and CTE remain valuable complementary tools, SBCE may be particularly advantageous for early disease detection and classification in very young children.

Push or balloon-assisted enteroscopy is another modality that allows direct visualization of small bowel lesions.^27^ In pediatric patients, double-balloon enteroscopy utilization has been reported, and it has been applied in diagnosing and managing pediatric IBD; the minimum body weight required for the procedure is approximately 11 kg.^28,29^ Although enteroscopy offers the distinct advantage of enabling histological assessment of deep small intestinal lesions, the lack of pediatric-specific enteroscopes and the technical difficulty of achieving complete small bowel visualization in children pose significant limitations. Consequently, SBCE may serve as a valuable alternative for both the diagnosis and longitudinal monitoring of VEO-IBD.

SBCE could fulfill various roles in the evaluation of VEO-IBD. Moreover, IBD-U accounts for approximately 30% of instances related to VEO-IBD.^30,31^ Therefore, SBCE may be advantageous in the differential diagnosis of CD by enabling a thorough examination of lesions within the small intestine. In the context of CD, the identification of lesions involving multiple sites advocates for the prompt initiation of biologic therapies, which may significantly impact treatment protocols.^32,33^ For the purpose of the differential diagnosis of monogenic IBD, including conditions such as CEAS, interleukin-10 deficiency, and XIAP deficiency, a holistic diagnostic strategy that encompasses the evaluation of the small intestine is essential.^34^ Considering these factors, SBCE may possess significant clinical value in the management of VEO-IBD.

There are several limitations to this study. First, the exact prevalence of gastrointestinal stenosis could not be determined, as patients with non-patent gastrointestinal tracts were excluded from the analysis. Second, SBCE findings were independently assessed by specialists at each participating institution, which may have introduced variability and a lack of centralized diagnostic consensus. Third, the evaluation of the clinical impact of SBCE was based on retrospective data and thus may have been influenced by the subjective judgment of attending physicians. To address these issues, prospective cohort studies are warranted to objectively assess the clinical impact of SBCE in the management of VEO-IBD.

In conclusion, our study suggests that the proper evaluation of gastrointestinal patency using PCs in patients with VEO-IBD can ensure the safe performance of SBCE. Although identifying small bowel lesions in patients with VEO-IBD and a smaller body size remains challenging, SBCE may serve as a valuable diagnostic modality.
